# Retrospective evaluation of venous phase contrast-enhanced computed tomography images in patients who developed pancreatic adenocarcinomas after treatment for nonpancreatic primary cancer

**DOI:** 10.1259/bjro.20200069

**Published:** 2021-07-05

**Authors:** Ryo Takaji, Yasunari Yamada, Ryuichi Shimada, Shunro Matsumoto, Tsutomu Daa, Yuichi Endo, Masafumi Inomata, Yoshiki Asayama

**Affiliations:** 1 Department of Radiology, Oita University Faculty of Medicine, Oita, Japan; 2 Department of Radiology, Oita Red Cross Hospital, Oita, Japan; 3 Department of Radiology, Almeida Memorial Hospital, Oita, Japan; 4 Department of Diagnostic Pathology, Oita University Faculty of Medicine, Oita, Japan; 5 Department of Gastroenterological and Pediatric Surgery, Oita University Faculty of Medicine, Oita, Japan

## Abstract

**Objectives:**

To clarify venous phase contrast-enhanced CT findings in early pancreatic adenocarcinomas by retrospectively evaluating CT images of pancreatic adenocarcinomas that developed during follow-up after treatment for non-pancreatic cancers.

**Methods:**

The study cohort comprised six patients who developed pancreatic adenocarcinomas between April 2005 and April 2020 during follow-up after treatment for non-pancreatic primary cancers. Two radiologists retrospectively evaluated CT images and reached consensus on previously reported CT findings that were suggestive of small pancreatic adenocarcinomas; namely pancreatic duct interruption and dilatation, pancreatic parenchymal atrophy, focal hypoattenuated areas, and appearance of cystic lesions. Time intervals between the first CT with these suggestive findings and the latest pre-operative CT were recorded. Doubling times were calculated in patients with hypoattenuated areas on initial CT scans.

**Results:**

Small (<10 mm) focal hypoattenuated areas with (*n* = 2) or without rim enhancement (*n* = 1) were identified on initial CT images of three patients. Pancreatic duct interruption and dilatation, pancreatic parenchymal atrophy, and cystic lesion were identified in two, one and one patient, respectively. Time intervals between initial and latest preoperative CT examination were 6–19 months (median, 14.5 months). Tumor doubling time according to CT findings was calculated as 46–407 days (median 106 days).

**Conclusion:**

Venous phase contrast-enhanced CT can provide findings that are suggestive of early pancreatic adenocarcinoma. Pancreatic phase contrast-enhanced CT should therefore be performed in patients with such findings with the aim of early detection of pancreatic adenocarcinoma.

**Advances in knowledge:**

Pancreatic adenocarcinoma can develop subsequently in patients with non-pancreatic malignancies. Patients with non-pancreatic cancers are often followed up with monophasic contrast-enhanced CT in venous phase timing. Venous phase contrast-enhanced CT can provide some findings suggestive of early pancreatic adenocarcinoma. Knowledge of these findings is important for early detection of pancreatic adenocarcinoma.

## Introduction

Pancreatic adenocarcinoma is one of the most lethal and difficult to treat malignant tumors.^1,2^ Surgical resection is the only curative treatment option; however, most patients have unresectable disease at the time of diagnosis.^3^ Furthermore, the survival rate of patients with pancreatic adenocarcinoma is better if small tumors are detected at an early stage.^4^ Therefore, early detection and precise diagnosis are very important for the management and prognosis of these patients. Multiphasic contrast-enhanced CT imaging is now considered the primary imaging modality for detection of pancreatic adenocarcinoma.^5^ Lu et al^6^ have stated that pancreatic phase images obtained beginning 40 sec after administration of contrast material show maximal tumor-to-pancreas contrast. Pancreatic adenocarcinomas usually appear as hypoattenuated lesions compared with the normal parenchyma in the pancreatic phase, whereas during the portal venous phase, these tumors are difficult to discern because of reduced contrast between the tumor and pancreatic parenchyma. However, patients with non-pancreatic cancers are often followed up with monophasic contrast-enhanced CT in venous phase timing performed after 70–100 sec from the initiation of intravenous contrast injection.^6^ Hence, small pancreatic adenocarcinoma could be missed in follow-up patients with other non-pancreatic cancer underwent surgery. Recently, some investigators have reported development of a subsequent pancreatic cancer in patients with non-pancreatic malignancies.^7–11^ The overall reported incidence of pancreatic cancer associated with other organ malignancies was 1.2–20%.^12^ Therefore, identification of the venous phase CT findings in early pancreatic adenocarcinomas is important for early diagnosis in patients who have previously been treated for non-pancreatic cancers. In this study, we retrospectively evaluated venous phase CT images that had been acquired during follow-up after treatment of previous nonpancreatic cancers with the aim of identifying incidental findings that suggest early pancreatic adenocarcinoma in such follow-up images.

## Methods and materials

### Patient selection

This retrospective study was approved by the ethics committee of our institution (Oita university hospital) and the requirement for informed consent was waived. The pathology and radiology databases and electric medical records of our institution were reviewed to identify patients with surgically proven pancreatic adenocarcinomas that had developed during follow-up after treatment of non-pancreatic cancer. Between April 2005 and April 2020, 147 consecutive patients with surgically proven pancreatic adenocarcinomas were identified.

Patients who met the following criteria were included in the study cohort: (a) surgically resected and pathologically diagnosed pancreatic adenocarcinoma; (b) had a previous non-pancreatic malignancy; (c) had undergone at least two venous phase contrast-enhanced CT examination; and (d) had no other pancreatic diseases.

The final study group comprised six patients (one male and five females; median age 69 years; age range, 61–86 years, median body weight 44.5 kg; body weight range, 30–48 kg). These patients’ previous nonpancreatic cancers were as follows: asynchronous triple cancer (colorectal, ureteral, and uterine cancers) (*n* = 1), colorectal cancer (*n* = 1), breast cancer (*n* = 1), gastric cancer (*n* = 1), malignant melanoma of left lower limb (*n* = 1) and hepatocellular carcinoma (*n* = 1). The hepatocellular carcinoma patient had undergone triple-phase contrast-enhanced CT and only the portal venous phase scan had been evaluated. The study cohort comprised only patients who undergone resection of a pancreatic adenocarcinoma that had been diagnosed definitively by pathological examination of the resected specimen.

### CT technique

Abdominal CT examinations were performed using a 64-section multidetector CT (MDCT) scanner (Aquilion CX TSX-101A/NA; Toshiba Medical Systems, Tokyo, Japan) or a 320-section MDCT scanner (Aquilion ONE TSX-301A/2A; Toshiba Medical Systems). The scanning parameters used for MDCT were as follows: 120 kVp, 200–400 mAs, rotation time of 0.5 s, pitch of 0.98 (64 detectors) and 0.6 (320 detectors), and section thickness of 1 mm with a 1 mm reconstruction interval. CT images were retrieved through a Picture Archiving and Communication System.

Venous phase contrast-enhanced images on follow-up for non-pancreatic cancer were obtained at 100 sec after starting the contrast-material infusion. A total of 100 ml of contrast-medium (Iopamiron 300; Bayer Schering Pharma, Berlin, Germany) was infused with a power injector at a rate of 3 ml s^−1^.

Triple-phase contrast-enhanced CT was performed for pancreatic adenocarcinoma staging. For triple-phase contrast-enhanced CT imaging, a total of 100 ml of contrast medium (Iopamiron 370; Bayer Schering Pharma, Berlin, Germany) was infused at a rate of 3 ml s^−1^ by means of a power injector. After unenhanced images had been acquired, all patients underwent pancreatic, portal venous, and equilibrium phase imaging. Each scan delay was determined using the automatic bolus-tracking method. The average scan delays from the injection of contrast material to the start of pancreatic, portal venous, and equilibrium phase imaging were 44, 75, and 158 s, respectively. The data sets obtained were sent to a computer workstation (Aquarius NetStation v. 1.2; TeraRecon, San Mateo, CA).

Reconstructed axial and multiplanar reconstruction images with 1 mm intervals were obtained using a computer workstation (Aquarius NetStation v. 1.2, Terarecon).

### Imaging interpretation

Two radiologists (R.S. and R.T., with 14 and 15 years of experience in abdominal imaging, respectively) reviewed all venous phase contrast-enhanced CT images obtained during follow-up after treatment of nonpancreatic cancer in all six patients. They evaluated the presence or absence of the following findings, which are all reportedly suggestive of small pancreatic adenocarcinomas: (1) pancreatic duct interruption and dilatation; (2) pancreatic parenchymal atrophy; (3) focal hypoattenuated areas; (4) pancreatic cystic lesions; and (5) enhanced rim.^13–18^ Ductal dilatation was defined as over 3 mm dilation of a main pancreatic duct or increase of more than 1 mm in main pancreatic duct diameter compared with latest surveillance CT. Pancreatic parenchymal atrophy was defined as atrophy of the parenchyma distal to a focal lesion or disproportional atrophy in the absence of any focal pancreatic lesions. Focal hypoattenuated areas were defined as localized areas of hypoattenuation in comparison with the normal pancreatic parenchyma. Time intervals between initial follow-up CT with findings suggestive of small pancreatic adenocarcinomas and latest pre-operative CT and doubling times were calculated from data obtained from hypoattenuated areas in CT images. In all of six cases, imaging findings suggestive of pancreatic adenocarcinoma were recognized on follow-up CTs, prompting triple-phase contrast-enhanced CT for staging of that pancreatic adenocarcinoma. Reconstructed axial and multiplanar reconstruction images from the staging CT were used to assess the pancreatic adenocarcinomas. Previous studies reported that triple phase (pancreatic, portal venous, and equilibrium phase) contrast-enhanced examination was useful for differentiating pancreatic adenocarcinoma from other pancreatic diseases, especially mass-forming pancreatitis^19^ and that biphasic (pancreatic and portal venous phase) contrast-enhanced examination was useful for staging of pancreatic adenocarcinoma.^20,21^ Therefore, triple-phase contrast-enhanced CT was performed for diagnosis and staging of pancreatic adenocarcinoma.

### Histopathological analysis

The post-operative pathology records of all six patients were retrospectively reviewed by a pathologist (T.D. with 31 years of experience). All pancreatic adenocarcinomas were proven by pathologic examination of surgically resected specimens. Tumor diameter, invasion into the anterior and posterior peripancreatic tissues, portal vein, and superior mesenteric vein, and lymph node metastases were also assessed in all patients.

## Results

**Figure 1.**
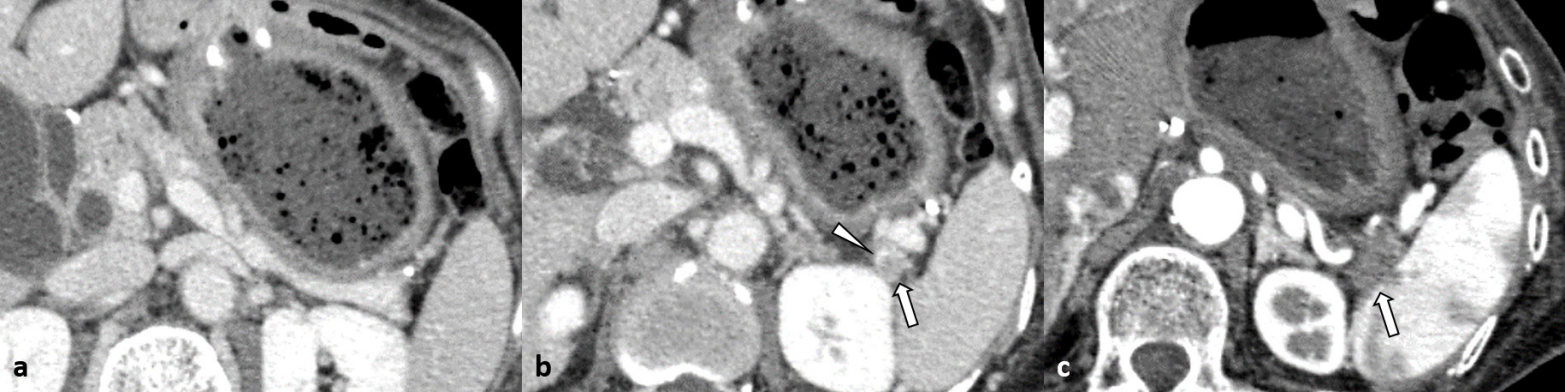
A 64-year-old female with pancreatic adenocarcinoma that developed after treatment for gastric cancer (Case 3) (**a**) Venous phase contrast-enhanced CT image obtained 26 months before the pancreatic surgery showing no abnormal findings suggestive of pancreatic adenocarcinoma. (**b**) Contrast-enhanced CT image obtained 14 months before the pancreatic surgery showing a small (8 mm diameter) hypoattenuation (arrow) lesion with an enhanced rim (arrowhead). (**c**) Triple-phase contrast-enhanced pancreatic phase CT image obtained immediately before pancreatic surgery showing an ill-defined hypoattenuated pancreatic tail mass (10 mm diameter, arrow). The pancreatic tail mass is invading the peripancreatic fat tissue and splenic hilum.

**Figure 2.**
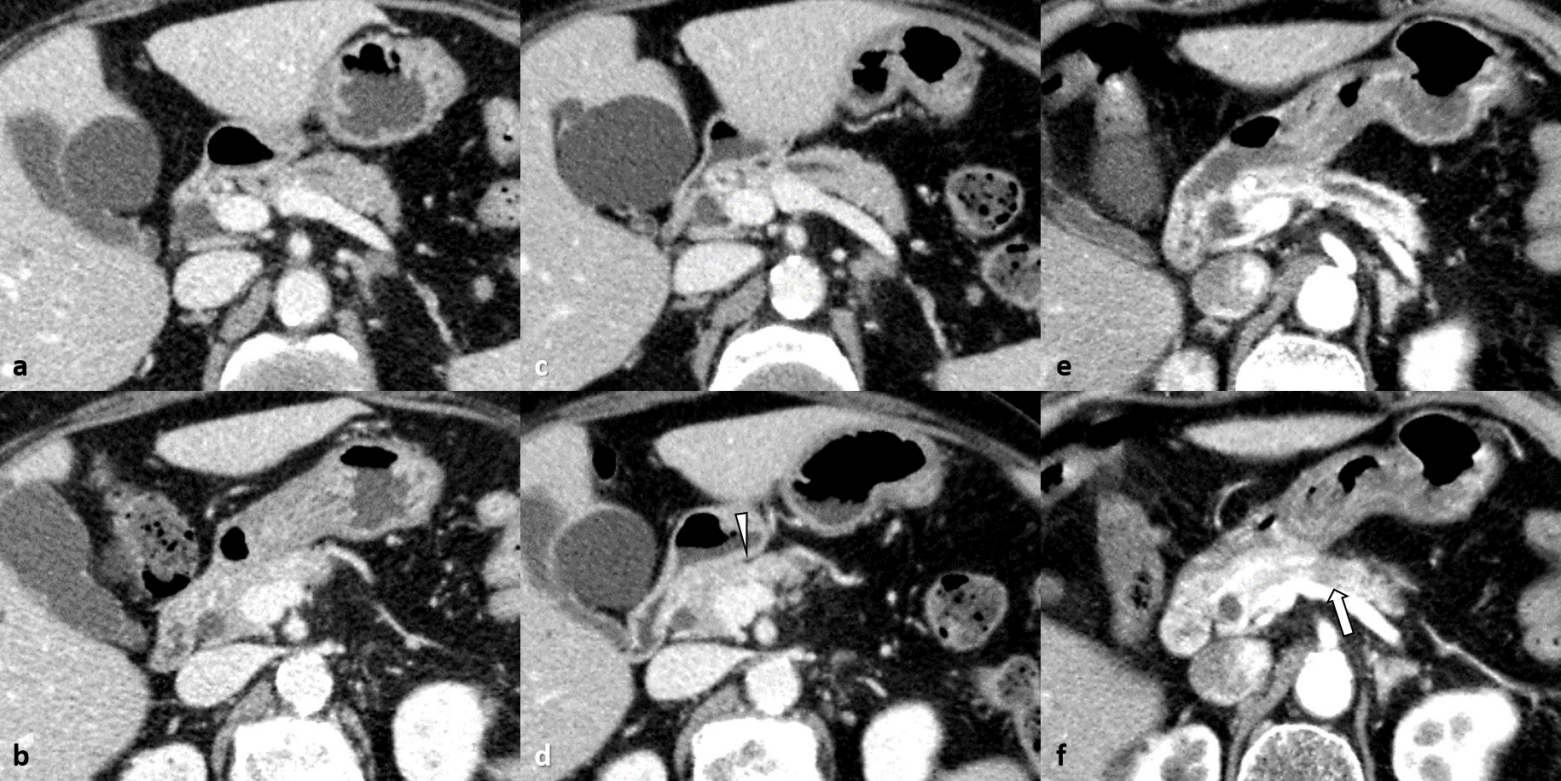
An 86-year-old female with pancreatic adenocarcinoma that developed after treatment for malignant melanoma of the left lower limb (Case 5) (a, b). Venous phase contrast-enhanced CT image obtained 24 months before the pancreatic surgery showing no abnormal findings suggestive of pancreatic adenocarcinoma. (**c, d**) Contrast-enhanced CT images obtained 15 months before the pancreatic surgery showing mild dilatation of the pancreatic duct. There is partial parenchymal atrophy (arrowhead) corresponding to the location of pancreatic duct interruption. (**e, f**) Triple-phase contrast-enhanced pancreatic phase CT image obtained immediately before pancreatic surgery showing a small hypoattenuated pancreatic body mass (10 mm diameter, arrow) and obvious upstream pancreatic duct dilatation. The pancreatic duct is interrupted by the mass, which is invading the peripancreatic fat tissue.

**Figure 3.**
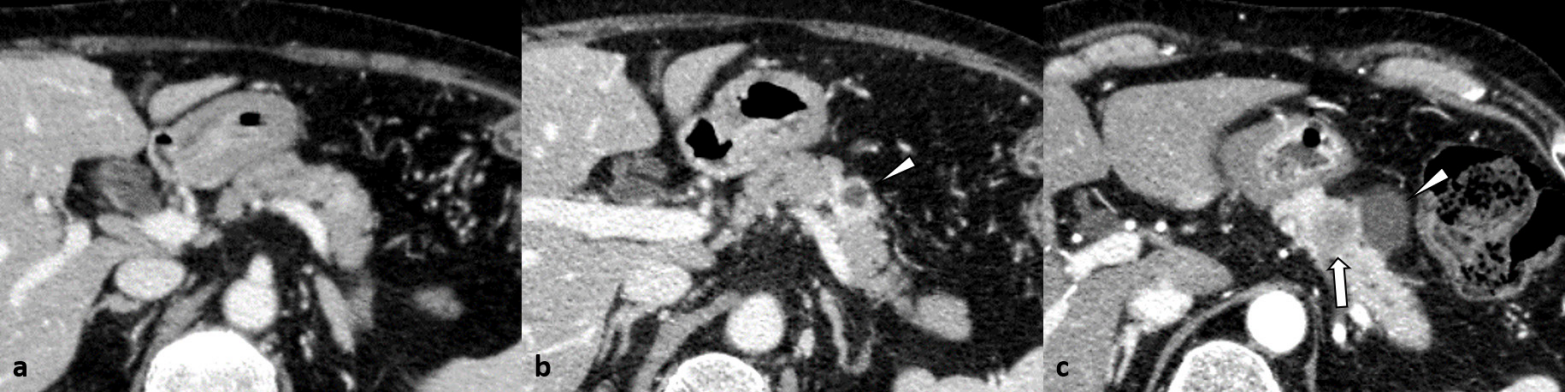
A 61-year-old female with pancreatic adenocarcinoma that developed after treatment for colon cancer (Case 6) (**a**) Venous phase contrast-enhanced CT image obtained 11 months before the pancreatic surgery showing no abnormal findings suggestive of pancreatic adenocarcinoma. (**b**) Contrast-enhanced CT image obtained 6 months before the pancreatic surgery showing a pancreatic cyst (11 mm diameter, arrowhead) with surrounding parenchymal enhancement in the pancreatic tail. (**c**) On triple-phase contrast-enhanced pancreatic phase CT image obtained immediately before pancreatic surgery showing that the pancreatic cyst (arrowhead) has increased in size. There is an obvious ill-defined hypoattenuated pancreatic tail mass (33 mm diameter, arrow) close to the pancreatic cyst. The solid pancreatic mass is invading the peripancreatic fat tissue.

Regarding initial CT findings suggestive of pancreatic adenocarcinoma (Table 1), small (≤10 mm) focal hypoattenuated areas were identified in three of six patients (50%) (Figure 1). Rim enhancement was observed in two of these three patients. Pancreatic duct interruption and dilatation (Figure 2), pancreatic parenchymal atrophy (Figure 2), and a cystic lesion (Figure 3) were identified in two (33%), one (17%) and one (17%) patient, respectively. The cystic lesion was pathologically confirmed to be a retention cyst. Time intervals between initial follow-up CT examination with findings suggestive of pancreatic adenocarcinoma and latest pre-operative CT examination were 6–19 months (median, 14.5 months) and follow-up CT examinations were performed 2–4 times during those intervals (median, 2.5 times). Doubling times as calculated from CT image data were 46–407 days (median, 106 days) in patients with focal hypoattenuated areas. The pancreatic adenocarcinomas in resected specimens were of diameter 12–55 mm (median, 18 mm). Local tumor invasion was detected pathologically in all six patients, comprising anterior tissue invasion in three patients, retropancreatic tissue invasion in five, neural plexus invasion in one, and duodenal invasion in one. Lymph node metastases were detected in three patients (50%); however, no distant metastases were identified in any of the patients. No focal lesions were identified on venous contrast-enhanced CT images obtained 6 months before pancreatic surgery in the patient with the largest diameter lesion (55 mm).

**Table 1.** CT findings suggestive of pancreatic adenocarcinoma on venous phase contrast enhanced CT images obtained during primary disease surveillance

Case	Location	CT findings suggestive of pancreatic cancer
1	Head	Small focal hypoattenuation^*a*^ (10 mm)
2	Head	Small focal hypoattenuation (5 mm)
3	Tail	Small focal hypoattenuation^*a*^ (8 mm)
4	Tail	Pancreatic duct dilatation/interruption
5	Body	Pancreatic duct dilatation/interruption, parenchymal atrophy
6	Tail	Cystic lesion (11 mm)

a Rim enhancement was also noted.

## Discussion

In this study, we retrospectively evaluated venous phase contrast-enhanced CT images of six patients with pancreatic adenocarcinomas that had developed during follow-up after treatment for non-pancreatic cancer. We found that several CT findings known to constitute possible early evidence of pancreatic adenocarcinoma (*e.g.* small focal hypo-attenuated areas with or without rim enhancement, pancreatic duct interruption and dilatation, pancreatic parenchymal atrophy, cystic pancreatic lesions)^13–18^ could have been detected on venous phase contrast-enhanced CT scans performed 6–19 months (median, 14.5 months) before the latest pre-operative CT. In three patients with small hypoattenuated areas on their initial CT images, tumor volume doubling times were 46–407 days (median 106 days).

Pancreatic cancer is associated with certain primary non-pancreatic cancers.^7–12^ Amin et al reported an increased risk of pancreatic cancer after gastric, colorectal, biliary and uterine cancers in patients aged over 65 years.^11^ It is expected that the incidence of development of pancreatic cancer after treatment of non-pancreatic cancer will increase in parallel with improved patient outcomes after treatment of the non-pancreatic primary cancers. Our study participants had various non-pancreatic cancers (namely gastric cancer, colorectal cancer, malignant melanoma, breast cancer and hepatocellular carcinoma) and one of them had asynchronous triple cancers (colorectal, ureteral, and uterine cancers). It may be important to pay particular attention to the possibility of pancreatic adenocarcinoma during surveillance after treatment of non-pancreatic primary cancers.

Ahn et al^13^ compared the CT findings in patients with delayed diagnosis of pancreatic cancers with those of a control group with non-pancreatic disease and chronic pancreatitis and found that focal low attenuated areas are commonly present in patients with early pancreatic cancer (sensitivity 75%, specificity 84%). Vernuccio et al^17^ retrospectively investigated missed CT evidence of pancreatic cancer, and also reported that focal low attenuation was the most common findings suggestive of early pancreatic cancer in all three of their patients. These investigators assessed pancreatic phase contrast-enhanced CT images or multiphasic contrast-enhanced CT images.

Lu et al^6^ have reported the usefulness of pancreatic phase imaging for detecting pancreatic adenocarcinoma. They found significantly greater differences between attenuation of pancreatic adenocarcinoma and parenchyma during the pancreatic phase than the hepatic phase (67 HU ± 19 vs 39 HU ± 16). The pancreatic phase is now considered optimal for detecting pancreatic adenocarcinoma. Fletcher et al^22^ have reported excellent sensitivities for tumor detection in the pancreatic phase (97%). Pancreatic adenocarcinomas can be clearly seen as hypoattenuated lesions compared with the normal parenchyma in the pancreatic phase, whereas identification of these tumors during the portal venous phase is not always easy.^7^ In our study participants, CT findings suggestive of early pancreatic adenocarcinoma were assessed only on venous phase contrast-enhanced CT images. Hence, focal hypoattenuated areas suggestive of pancreatic adenocarcinoma were identified on initial CT images in only three of the six patients (50%). It is possible that small areas of hypoattenuation would have been detected more frequently if pancreatic phase contrast-CT had been performed. Small pancreatic adenocarcinoma occasionally have enhanced rims in the portal venous to equilibrium phase.^18^ In our study, enhanced rims were identified in two of the three patients with hypoattenuated areas. Small focal areas of hypoattenuation with enhanced rims on venous phase contrast-enhanced CT images may be an important indicator of early pancreatic adenocarcinoma. Several investigators have reported that pancreatic duct dilatation is the earliest indicator of pancreatic cancer.^23,24^ Amin et al^11^ also reported that pancreatic duct interruption and dilatation can indicate pancreatic cancer in patients without a mass; however, the sensitivity was relatively low (dilation; 50%, interruption; 45%). Furthermore, Yamao et al^14^ reported that the presence of partial pancreatic parenchymal atrophy and/or pancreatic duct abrupt stenosis on CT images is highly suggestive of small (≤10 mm) pancreatic adenocarcinomas. In our study, pancreatic duct dilatation without a pancreatic mass was identified on initial venous phase contrast-CT in two of the six patients (33%) and partial pancreatic parenchymal atrophy surrounding pancreatic duct interruption was identified in one patient (16%). Tada et al^16^ reported that pancreatic cystic lesions such as intraductal papillary mucinous neoplasm and simple cysts are associated with a high risk of pancreatic adenocarcinoma. One patient (17%) in our study developed a pancreatic adenocarcinoma after having a pathologically diagnosed retention cyst. Strong parenchymal enhancement surrounding the cyst was noted in this patient. This finding may have reflected blockage of pancreatic juice drainage caused by a small pancreatic adenocarcinoma. Pancreatic duct interruption and dilatation, partial pancreatic atrophy, and pancreatic cysts on venous phase contrast-enhanced CT images are considered to be suggestive of pancreatic adenocarcinoma.

It is difficult to assess the natural history of pancreatic adenocarcinoma. Ahn et al^25^ evaluated 100 patients with pathologically proven pancreatic cancers who had undergone at least two CT examinations prior to the final diagnosis and reported that small pancreatic adenocarcinomas (≤20 mm) on initial CT grow slowly and take significantly longer to develop distant metastases than do larger pancreatic adenocarcinomas (20 mm<). According to these authors, the tumor volume doubling time for pancreatic adenocarcinoma ranged from 20.0 to 976.8 days (mean 132.2 days). In the present study, all three small hypoattenuated areas on initial CT that could have been suspected of being pancreatic adenocarcinomas were less than 20 mm in diameter (5–10 mm, median 8.5 mm). Tumor volume doubling time as calculated from CT data was shorter than previously reported (46–407 days, median 106 days). Tumor diameters measured on resected specimens were 12–55 mm (median 18 mm). Local tumor invasion was identified in all cases (100%). In Case 6, the patient with a cystic lesion, the tumor grew rapidly to 55 mm in diameter in the 6 months before pancreatic surgery. Hence, when findings suspicious of pancreatic adenocarcinoma (*e.g.* small areas of hypoattenuation, pancreatic duct dilatation and interruption, parenchymal atrophy, and pancreatic cysts) are detected during post-operative follow-up of patients with non-pancreatic primary cancers, prompt action regarding a possible pancreatic adenocarcinoma is imperative.

This study had several limitations. First, its retrospective nature may have been a source of selection bias. Second, it was a small study. Third, the reviewers were aware of the diagnosis. However, the purpose of our study was to investigate early evidence of pancreatic adenocarcinoma on venous phase contrast-enhanced CT images. Further larger prospective studies are required to confirm the indicators of pancreatic adenocarcinoma on venous contrast-enhanced CT.

In summary, small hypoattenuated areas with or without rim enhancement, pancreatic duct interruption and dilatation, focal parenchymal atrophy, and cystic lesions on venous phase contrast-enhanced CT images are suggestive of early pancreatic adenocarcinoma.
